# Demoralization predicts suicidality in patients with cluster headache

**DOI:** 10.1186/s10194-021-01241-7

**Published:** 2021-04-20

**Authors:** Brian B. Koo, Ahmed Bayoumi, Abdalla Albanna, Mohammed Abusuliman, Laura Burrone, Jason J. Sico, Emmanuelle A. D. Schindler

**Affiliations:** 1 Department of Neurology, Yale University School of Medicine (SOM), PO Box 208018, New Haven, CT 06520-8018 USA; 2 Department of Neurology, Veterans Affairs Connecticut Healthcare System, West Haven, CT USA; 3 Veterans Affairs Headache Centers of Excellence: Research Hub, West Haven, CT USA; 4 Center for NeuroEpidemiologic and Clinical Neurological Research, Yale University School of Medicine, New Haven, CT USA

**Keywords:** Cluster headache, Suicide, Depression, Demoralization, Suicidal ideation

## Abstract

**Objective:**

To determine the frequency of suicidal ideation and assess suicide risk in cluster headache (CH) patients compared to matched controls without CH in this observational case-control study.

**Background:**

CH is characterized by recurrent intolerable attacks of unilateral retro-orbital pain, which can cause disability, depression, and desperation. CH has been linked to suicide since its early descriptions by B.T. Horton; however, there is relatively little empiric data showing the association between suicidality and CH, especially in the context of other psychological phenomena, such as depression and demoralization.

**Methods:**

CH and control participants were recruited through community and CH patient group advertisements. CH diagnosis was confirmed using the International Classification of Headache Disorders, 3rd edition diagnostic criteria for CH. Lifetime suicidal ideation and suicide risk were assessed using the Suicidal Behavior Questionnaire-revised and the Columbia Suicide Severity Rating Scale. The Brief Lifetime Depression Scale evaluated lifetime depression. Demoralization was assessed using the Diagnostic Criteria for use in Psychosomatic Research – Demoralization and the Kissane Demoralization Scale. Forward stepwise logistic regression determined the odds of suicidal ideation.

**Results:**

One hundred CH and 135 control participants were comparable for age, sex, race, income, and marital status. Significantly more CH than control participants had lifetime active suicidal ideation (47.0% vs. 26.7%; *p* = 0.001), high suicide risk (38.0% vs. 18.5%; *p* = 0.0009), lifetime depression history (67.0%% vs. 32.6%; *p* < 0.00001), and demoralization (28.0% vs. 15.6%; *p* = 0.02). The odds of lifetime suicidal ideation were higher in those with CH (odds [95% confidence interval]; 2.04 [1.08,3.85]), even after accounting for depression and demoralization. In CH, suicidal ideation was associated with demoralization (6.66 [1.56,28.49]) but not depression (1.89 [0.66,5.46]).

**Conclusions:**

Lifetime suicidal ideation and high suicide risk are prevalent in CH sufferers, and its likelihood is dependent on the presence of demoralization.

## Introduction

The particular characteristics of cluster headache (CH) impart a marked burden on its patients. CH is the most common trigeminal autonomic cephalalgia, characterized by recurrent disabling attacks of unilateral retro-orbital pain accompanied by ipsilateral autonomic symptoms such as lacrimation, ptosis, nasal congestion and/or facial sweating. Headache attacks recur over periods of weeks to months separated by attack-free remission periods lasting months to years; this episodic form of CH comprises approximately 80% of cases. The remaining one-fifth of cases have chronic CH, wherein attacks recur for at least a year with no more than 3 months of attack-free reprieve [1, 2]. Apart from the idiosyncratic time course of attacks, several unique features distinguish CH from other headache disorders, such as restlessness during attacks and the characteristic intensely excruciating pain, which is rated more severe than even childbirth or nephrolithiasis [3]. These recurrent intensely painful headache attacks impose a significant personal and economic burden on CH sufferers, as nearly one-fifth report losing a job due to CH and a similar number are homebound for days at a time at least once annually [4].

These unrelenting paroxysms of intense pain and associated personal and economic burdens of disease are a setup for poor mental health. Indeed major depressive episodes within a lifetime are more than two-and-a-half times more likely to occur in CH sufferers than those without CH [5]. In fact, CH’s link to suicide, the most severe consequence of depression, was first described by the American neurologist, B.T. Horton, in 1939 as he wrote, “Our patients were disabled by … pain … so severe that several had to be constantly watched for fear of suicide.” [6] In CH, however, it is unclear whether suicide or suicidal ideation (SI) is a consequence of depression or rather a perceived solution to end profound suffering in a desperate individual. This latter mental state of desperation may not be adequately captured by an assessment of depression alone. Demoralization is a well characterized psychological state in which individuals suffer a profound sense of hopelessness, an inability to cope with problems, and feelings of being trapped [7]. While depression and demoralization share some features, such as feelings of hopelessness and guilt, in depression, the appropriate course of action is known, yet motivation is lacking, whereas in the demoralized state, there is uncertainty as to the appropriate course of action [8]. Thus, demoralization may better characterize the feeling of desperation felt in CH sufferers than depression [9].

Studies have provided evidence that SI is common among persons with CH. In a large survey of 1134 individuals with CH, suicidal thoughts were found in 55% [4]. More recently, a high prevalence of suicidality was found among individuals comprising the Korean Cluster Headache registry in which more than one-third reported having active SI during a cluster attack and nearly 10% had a plan to commit suicide [10]. These studies introduced empirical evidence linking CH to SI; however, interpretation was somewhat limited as neither a control group nor assessment of additional sociodemographic and psychological factors were included. Comparison to a matched control group allows for evaluation of the degree to which high suicidality is attributed to CH, as depression can be assessed in both groups.

Given the notable association between CH and SI, and in light of the paucity of rigorously measured evidence to support this association, we aimed to study whether or not suicidal thought and behavior is increased among CH sufferers. Moreover, we aimed to determine the frequency of lifetime SI and suicide risk in patients with CH and sociodemographically matched controls. At the same time, we included assessments of medical disease, depression, and demoralization to discriminate which clinical and psychological factors contributed to suicidality. We hypothesized that SI and suicide risk would be significantly more common in CH sufferers than matched controls, even after considering sociodemographics and clinical and psychological history, and that demoralization in addition to depression would be associated with suicidality.

## Methods

### Participants, recruitment, & CH assessment

We recruited patients with CH from October 2019 to January 2021 using advertisements on the Clusterbusters.org website, Facebook CH support groups, and through email solicitation to individuals identifying as having CH on Research Match, a national registry of individuals interested in participating in clinical research. Control participants were recruited through email solicitation through Research Match and the Yale Research Subject Registry. The recruitment email indicated that the aim of the study was to investigate lifestyle factors and their relation to CH and also compare the presence of such factors in individuals with and without CH. All recruitment materials, emails and advertisements avoided mentioning depression or suicide. At the end of each survey, as sensitive questions about suicide were asked, contact information was provided for the National Suicide Prevention Lifeline and Yale Crisis Services.

The entire study was conducted using the electronic survey tool, Yale Qualtrics system, including both participant screening and questionnaire completion. The study was anonymous and participants were not assessed in person by study personnel. Prior to answering questions, a full description of the study methods and risks was provided via Qualtrics. The anonymous nature of the study was highlighted and thus by choosing to proceed, potential subjects expressed their comprehension of the study, providing their informed consent as approved by the Yale University Ethics Board. Initial screening included inclusion/exclusion criteria, and based upon these criteria, potential participants were deemed eligible and allowed to proceed to the questionnaires or deemed ineligible in which case the survey and their participation ended. Inclusion criteria were age between 18 and 89 years and ability to read and write in English. Exclusion criteria were having other neurological conditions that commonly cause headache, such as idiopathic intracranial hypertension, brain tumors, or a brain lesion (e.g., hemorrhage, infection). Concomitant headache disorders such as migraine were not exclusionary.

Potential participants were screened through the online survey for CH, using screening questions consistent with International Classification of Headache Disorders 3rd edition (ICHD-3) diagnostic criteria for CH [2]. A person was classified in the CH group if they fulfilled the following criteria: answered “Yes” to the two questions in item (1): (1) “Have you had severe or very severe attacks of unbearable pain in the region of the eye or temple on one side?” and “Does the ache always appear on the same side of the head?” and attack characteristics also fulfilled criteria (2), (3), and (4): (2) attack duration 15 to 180 min; (3) frequency of attacks between one every other day to eight per day; and (4) there was either autonomic symptoms ipsilateral to headache in the form of lacrimation, nasal congestion/rhinorrhea, eyelid edema, facial sweating, miosis, or ptosis, or restlessness or agitation, or both. Those with CH were subclassified as having episodic CH, if there had been at least two cluster periods lasting 7 days to 1 year, separated by attack-free periods of at least 3 months, or as chronic CH if attacks occurred without remission periods or with remission lasting < 3 months. Participants were allocated to the control group if they answered ‘No’ to “Have you had severe or very severe attacks of unbearable pain in the region of the eye or temple on one side?” and endorsed that they did not have CH.

Quality of life specific to CH was determined using the CH Quality of Life Questionnaire (CH-QOL), a 28-item instrument which assesses certain negative feelings or behavioral concessions of CH (e.g., felt aggressive, avoided leaving home, felt bad about yourself) [11]. The questionnaire assesses the degree to which common social, occupational, and everyday life activities were negatively affected by CH. Answer choices were 0 = never, 1 = occasionally, 2 = sometimes, 3 = often, or 4 = always. Scores ranged from 0 to 112.

### Assessment of depression, demoralization, and suicidality

Depression, panic disorder, and bipolar disorder were assessed by self-report and major depression history was assessed by validated questionnaire. To determine a history of depression, panic disorder, or bipolar disorder, subjects were asked to select from a menu of medical problems which included depression. To assess lifetime depression history, we used the Brief Lifetime Depression Scale (BLDS), which conforms to the Diagnostic and Statistical Manual of Mental Disorders-V criteria for major depressive disorder. The scale inquires whether in one’s lifetime, there was a period of at least 2 weeks in which there were ≥ 5 of the following symptoms with at least one being depressed mood or anhedonia: (1) feeling down, depressed, or hopeless; (2) having little interest in doing things; or (3) were less able to enjoy things. If answering “Yes” (1), (2), or (3), they were asked if they had experienced (4) sleep problems, (5) fatigue, (6) appetite changes, (7) feelings of being a failure, (8) concentration problems, (9) psychomotor retardation, or (10) passive SI [12, 13]. The use of BLDS and the assessment of major depression in one’s lifetime was crucial as suicidality was also assessed within one’s lifetime.

To assess demoralization, the Diagnostic Criteria for use in Psychosomatic Research – Demoralization (DCPR-D) criteria and the Kissane Demoralization Scale (KDS) were used. The DCPR-D questionnaire included the following questions pertaining to the preceding 12 months: (1) Do you feel you have failed to meet your expectations or those of other people?; (2) Is there an urgent problem you feel unable to cope with?; (3) Do you experience feelings of helplessness, hopelessness, and/or giving up? If the participant answered “Yes” to the prior questions, the following was asked: (4) If you have experienced feelings of helplessness, hopelessness, and/or giving up, did your state of feeling exceed a month? DCPR-D criteria were met when positive responses to questions (3) and (4) were combined with positive responses to either question (1) or (2) [14]. The KDS consists of 24 items assessing the frequency over the past 2 weeks that participants had certain negative feelings (0–4; never to all the time; range 0-96) (e.g., “I feel hopeless”; “No one can help me.”; “I feel trapped by what is happening to me.”). Demoralization was defined as KDS score ≥ 42 (overall cohort mean plus 1 SD of cohort) [15]. Demoralization in total was defined as meeting DCPR-D criteria or KDS ≥ 42.

Suicidality, including passive and active SI and suicide attempts, were assessed using the Suicidal Behavior Questionnaire-revised (SBQ-R) and the Columbia Suicide Severity Rating Scale (C-SSRS). The SBQ-R consists of four questions: (1) Have you ever thought about or attempted to kill yourself? (1 = never, 2 = just a brief passing thought, 3 = have had a plan at least once to kill myself, 4 = have attempted to kill myself); (2) How often have you thought about killing yourself in the past year? (1 = never, 2 = rarely, 3 = sometimes, 4 = often, 5 = very often); (3) Have you ever told someone that you were going to commit suicide, or that you might do it? (1 = no, 2 = yes at one time, 3 = yes more than once); (4) How likely is it that you will attempt suicide someday? (0 = never, 1 = no chance at all, 2 = rather unlikely, 3 = unlikely, 4 = likely, 5 = rather likely, 6 = very likely). Risk of suicidal behavior was considered high if total SBQ-R score was ≥7, a standardly used cutoff in non-clinical samples [16]. Having had a suicide plan and having attempted suicide in one’s lifetime was assessed by question (1). The C-SSRS was used to further assess SI, using the following questions: (1) Have you wished you were dead or wished you could go to sleep and not wake up?, assessed passive SI; (2) Have you actually had any thoughts of killing yourself (in your lifetime)?, assessed active SI [17].

### Assessment of covariates

Collected demographic information included age, sex, race, marital status, education, employment, and income. Lifetime drug and alcohol history and current antidepressant medication use were also noted. Completion of college was noted for education. Regarding marital status, those currently married constituted one group compared to single, divorced, separated, or widowed individuals. Participants with middle or high income were grouped separately from those with low income (monthly income ≤$3000). Regular drug use was defined as a period 1 year or more in which recreational drugs were used weekly. Regular alcohol use was defined as a period of 1 year or more in which alcohol was consumed ≥3 days per week with ≥2 drinks per day.

### Statistical analysis

Sample size was calculated using a 10% prevalence of high suicide risk found in a previous study [18] and a best guess prevalence in CH of 25%, which would require a sample size of 100 in each group given a 0.05 *p*-value and 80% power. Characteristics were compared between CH and control groups using chi-squared for categorical and student’s t-tests for continuous variables. For logistic and multivariable regression models, the primary outcomes were lifetime active or passive SI from the C-SSRS and SBQ-R score. Models were carried out in the overall cohort and in the CH only cohort. Forward stepwise regression was used. Confounding variables (variables of interest) were entered into the final model based upon their association in univariate analysis (*p*-value ≤0.10) with SI or SBQ-R score. Variables associated with suicide in previous population studies (e.g. age, sex, race, marital status, education, income, and drug/alcohol abuse) were also entered in the model [19]. Statistical analysis was performed using the R statistical package 4.0.3 (Auckland, New Zealand).

## Results

### Characteristics of cluster headache and control groups

One-hundred CH (53 men, 53.0%) and 135 control (77 men, 57.0%) participants were included in the study. Eighteen CH and 25 control participants started but did not complete suicide questionnaires and were excluded. The excluded individuals with CH (age 44.8 ± 14.5, 10 male, 66.7% episodic) were similar in socio-demographic characteristics to included CH participants. The excluded individuals without CH were similar to included control participants. CH and control groups were comparable for age, sex, Caucasian race, current marital status, current employment, and income (Table 1). A significantly lower percentage of CH participants completed college than did controls (78.0% vs. 92.6%; *p* = 0.001); however, both groups consisted predominantly of individuals who completed college. Lifetime history of regular alcohol use was similar between the groups; however, significantly more CH participants had a lifetime history of regular drug use. There was no difference in the past regular use of drugs, including marijuana, opioids, lysergic acid diethylamide, cocaine, or heroin, between groups, except for psychedelic mushrooms, which were used regularly (at one point) more frequently by persons with CH (12.0% vs. 3.0%; *p* = 0.007). In general, both groups were medically healthy, but significantly more controls had self-reported hypertension and diabetes mellitus, albeit at relatively low rates.

**Table 1:** Demographics and Medical History of Cluster Headache patients and controls

Demographics	Overall Cohort (***n*** = 235)	Cluster (***n*** = 100)	Controls (***n*** = 135)	***p***-value^a^
**Age (mean ± SD)**	46.1 ± 14.9	45.5 ± 12.3	46.5 ± 16.6	0.57^b^
**Sex (male;** ***n*, %)**	130/235 (55.3)	53/100 (53.0)	77/135 (57.0)	0.54^c^
**Race (Caucasian;** ***n*, %)**	221/235 (94.0)	94/100 (94.0)	127/135 (94.1)	0.98^c^
**Currently married;** ***n*, %)**	138/235 (58.7)	63/100 (63.0)	75/135 (55.6)	0.25^c^
**Higher Education (finished college;** ***n*, %)**	203/235 (86.4)	78/100 (78.0)	125/135 (92.6)	0.001^c^
**Economic**^d^				0.44^c^
**High/middle (n, %)**	166/235 (70.6)	68/100 (68.0)	98/135 (72.6)	
**Low**	69/235 (29.4)	32/100 (32.0)	37/135 (27.4)	
**Employed (n, %)**	196/235 (83.4)	80/100 (80.0)	116/135 (85.9)	0.23^c^
**Regular Alcohol Use**^e^ **(n, %)**	18/235 (4.7)	7/100 (7.0)	11/135 (8.1)	0.57^c^
**Regular Drug Use**^f^ **(n, %)**	48/235 (16.7)	32/100 (24.6)	16/135 (10.2)	0.001^c^
**Psychedelic Mushroom (n, %)**	16/235 (5.5)	12/100 (12.0)	4/135 (3.0)	0.007^c^
**Marijuana (n, %)**	99/235 (42.1)	45/100 (45.0)	54/135 (40.0)	0.44^c^
**Hypertension (n, %)**	39/235 (16.6)	10/100 (10.0)	29/135 (21.5)	0.02^c^
**Diabetes Mellitus (n, %)**	15/235 (6.4)	2/100 (2.0)	13/135 (9.6)	0.02^c^

^a^Comparing Cluster headache persons to Controls; superscript letter after *p*-value indicates type of test used; ^b^Student’s t-test; ^c^Chi Squared test; ^d^For the economic variable, High ≥ $6000 monthly income; Middle = $3001 to $6000 monthly income; Low ≤ $3000 monthly income; ^e^For History of Regular Alcohol Use: Period of one year when 3 or more drinks at least 3 times per week; ^f^For History of Regular Drug Use: Period of one year when drugs were used weekly, referenced to those that had less than this amount of use

### Mental health & suicidality

Persons with CH had a significantly greater mental health burden than controls (Table 2). Self-reported panic disorder and bipolar disorder were significantly greater in CH. While neither self-report of having depression nor report of taking an antidepressant medication were different between groups, more than two-thirds of CH sufferers had a lifetime episode of depression, more than double that of controls (67.0% vs. 32.6%; *p* < 0.00001).

**Table 2:** Mental Health and Suicidality in Cluster Headache patients and controls

Demographics	Overall Cohort (***n*** = 235)	Cluster Headache (***n*** = 100)	Controls (***n*** = 135)	***p***-value^a^
**Panic Disorder**	16/235 (6.8)	12/100 (12.0)	4/135 (3.0)	0.007^c^
**Bipolar**	19/235 (8.1)	15/100 (15.0%)	4/135 (3.0)	0.0008^c^
**Depression (n, %)**^d^	45/235 (19.1)	18/100 (18.0)	27/135 (20.0)	0.70^c^
**Current Antidepressant (n, %)**	46/235 (19.6)	17/100 (17.0)	29/135 (21.5)	0.39^c^
**Lifetime Depression (n, %)**^e^	111/235 (47.2)	67/100 (67.0)	44/135 (32.6)	< 0.00001^c^
**Demoralized**^f^	49/235 (20.9)	28/100 (28.0)	21/135 (15.6)	0.02^c^
**KDS**	22.9 ± 18.6	28.3 ± 19.7	18.9 ± 16.8	0.0002^b^
**Lifetime Suicide plan (n, %)**^g^	46/235 (19.6)	25/100 (25.0)	21/135 (15.6)	0.07^c^
**Lifetime Suicide attempt (n, %)**^g^	17/235 (7.2)	8/100 (8.0)	9/135 (6.7)	0.70^c^
**SBQ-R Score**	5.3 ± 2.9	6.1 ± 3.1	4.7 ± 2.6	0.0003^b^
**SBQ-R High Risk Suicide**	63/235 (26.8)	38/100 (38.0)	25/135 (18.5)	0.0009^c^
**C-SSRS: Passive Suicidal Ideation**	146/235 (62.1)	59/100 (59.0)	47/135 (34.8)	0.0002^c^
**C-SSRS: Active Suicidal Ideation**	83/235 (35.3)	47/100 (47.0)	36/135 (26.7)	0.001^c^

*SBQ-R* Suicidal Behavior Questionnaire- Revised, *C-SSRS* Columbia Suicide Severity Rating Scale, *KDS* Kissane Demoralization Scale; ^a^Comparing Cluster headache persons to Controls; superscript letter after p-value indicates type of test used; ^b^Student’s t-test; ^c^Chi Squared test; ^d^Depression was self-report; ^e^Lifetime Depression was determined by Brief Lifetime Depression Scale; ^f^Demoralized was defined as fulfilling Demoralization diagnostic criteria for use in Psychosomatic research or KDS score ≥ 42;^g^Lifetime suicide plan and lifetime suicide attempt was determined by BSQ-R

Lifetime suicidality was also significantly more common among CH sufferers. From the SBQ-R, marginally more persons with CH than controls had a plan for suicide in their lifetime (*p* = 0.07), but lifetime suicide attempts did not differ between the groups. Those found to be at high suicide risk, as evidenced by an SBQ-R score ≥ 7, were significantly more common, 38% vs. 18.5% (*p* = 0.0009), in the CH than control group. Using the C-SSRS, both passive and active SI were significantly more frequent in CH, with nearly two-thirds having passive SI and nearly half having active SI in the CH group. Demoralization was significantly more common in the CH group (28.0% vs. 15.6%; *p* = 0.02). This was also reflected in significantly higher KDS scores in the CH group.

Linear regression models showed that lifetime regular drug use, lifetime depression and being demoralized were associated with higher SBQ-R score, one point higher for regular drug use and lifetime depression and nearly 3 points higher for being demoralized (Table 3). Having CH was marginally associated with SBQ-R score. In a sensitivity analysis, removing regular drug use history from the model, having CH was significantly associated with SBQ-R score (0.7 [0.1, 1.4]; *p* = 0.049), likely because CH sufferers were more likely to have had regular drug use.

**Table 3:** Associations of Suicidal Ideation or High Suicide Risk in the Overall Cohort

Outcome: Passive or Active Suicidal Ideation		
Variable	Odds Ratio (95% CI)	*p*-value
Cluster Headache^a^	2.04 (1.08, 3.85)	0.03*
Lifetime depression^b^	2.30 (1.18, 4.46)	0.01*
Demoralized^c^	3.33 (1.39, 8.04)	0.007*
Age (per 1 year)	1.00 (0.98, 1.02)	0.92
Male sex^d^	1.29 (0.65, 2.54)	0.47
Caucasian^e^	0.50 (0.14, 1.78)	0.29
Currently Married^f^	0.54 (0.27, 1.09)	0.09
Completed College^g^	0.52 (0.21, 1.28)	0.15
High/Middle Income^h^	1.03 (0.48, 2.23)	0.94
Regular Drug Use history^i^	1.35 (0.57, 3.19)	0.50
Regular Alcohol Use history^j^	0.99 (0.33, 3.01)	0.99
Outcome: SBQ-R Score (Range 3-18)		
Variable	SBQ-R Score points (95% CI)	*p*-value
Cluster Headache^a^	0.6 (0.1, 1.2)	0.12
Lifetime depression^b^	1.0 (0.2, 1.7)	0.01*
Demoralized^c^	2.8 (1.9, 3.7)	< 0.0001*
Age (per 10 year)	0.1 (−0.1, 0.4)	0.26
Male sex^d^	0.0 (−0.7, 0.7)	0.95
Caucasian^e^	−0.4 (−1.8, 0.9)	0.55
Currently Married^f^	− 0.4 (−1.2, 0.3)	0.25
Completed College^g^	−0.3 (−1.3, 0.6)	0.53
High/Middle Income^h^	−0.5 (− 1.3, 0.3	0.19
Regular Drug Use history^i^	1.0 (0.1, 1.9)	0.03*
Regular Alcohol Use history^j^	−0.1 (−1.2, 1.1)	0.92

Reference groups: ^a^Cluster headache: referent controls; ^b^Lifetime depression: referent no depression; ^c^Demoralization: defined as fulfilling demoralization diagnostic criteria for use in psychosomatic research or KDS score ≥ 42; ^d^Male sex: referent female; ^e^Caucasian: referent other races; ^f^Currently married: referent currently single, divorced, widowed; ^g^Completed college: referent did not complete college; ^h^High/middle income: referent low income (< $3000 monthly); ^i^Regular drug use: period of one year when drugs were used weekly, referenced to those that did not have such a period; ^j^History of regular alcohol use: period of one year when 3 or more drinks at least 3 times per week, referenced to those who did not have such a period

* = *p*<0.05

Logistic regression showed that the odds of having passive or active SI in one’s lifetime was more than two-fold in those with CH compared to controls, even while statistically adjusting for lifetime depression, which was also significantly associated with SI odds, and for age, sex, race, marital status, education, income, and drug and alcohol use history, which were not associated with the odds of SI (Table 3). Demoralization was associated with the highest odds of lifetime SI; its presence increased the odds of lifetime SI more than three-fold.

### Cluster headache group

Among the 100 CH sufferers, 56 had episodic and 44 had chronic CH. Episodic and chronic CH sufferers were similar in age, sex, and race (Table 4). Groups scored similarly on the CH-QOL scale, although scores were marginally higher in chronic CH sufferers, indicating a lower quality of life. Demoralization was significantly more common in the chronic CH group (40.9% vs. 17.9%; *p* = 0.01), which was reflected in the significantly higher KDS scores among chronic CH sufferers. Comparing chronic to episodic CH sufferers, having had a suicide plan in one’s lifetime was significantly more frequent, and having had a suicide attempt was marginally more common in chronic CH.

**Table 4:** Demographics and Mental Health in Cluster Headache Patients

Demographics	All Cluster (***n*** = 100)	Episodic (***n*** = 56)	Chronic (***n*** = 44)	***p***-value^a^
**Age (mean ± SD)**	45.5 ± 12.3	43.9 ± 12.8	47.4 ± 11.5	0.14^b^
**Sex (male; n, %)**	53/100 (53.0)	30/56 (53.6)	23/44 (52.3)	0.54^c^
**Race (Caucasian; n, %)**	94/100 (94.0)	53/56 (94.6)	41/44 (93.2)	0.76^c^
**Lifetime Depression (n, %)**^d^	67/100 (67.0)	34/56 (60.7)	33/44 (75.0)	0.13^c^
**Demoralized**^e^	28/100 (28.0)	10/56 (17.9)	18/44 (40.9)	0.01^c^
**KDS**	28.3 ± 19.7	23.3 ± 17.8	34.6 ± 20.4	0.005^c^
**Lifetime Suicide plan (n, %)**^f^	46/235 (19.6)	9/56 (16.1)	16/44 (36.4)	0.02^c^
**Lifetime Suicide attempt (n, %)**^e^	8/100 (8.0)	2/56 (3.6)	6/44 (13.6)	0.07^c^
**CH-QOL**	63.1 ± 22.0	60.5 ± 21.9	66.4 ± 22.1	0.18^c^

*KDS* Kissane Demoralization Scale, *CH-QOL* Cluster Headache Quality of Life Questionnaire score ^a^Comparing Cluster headache persons to Controls; superscript letter after p-value indicates type of test used; ^b^Student’s t-test; ^c^Chi Squared test; ^d^Lifetime Depression was determined by Brief Lifetime Depression Scale; ^e^Demoralized was defined as fulfilling Demoralization diagnostic criteria for use in Psychosomatic research or KDS score ≥ 42; ^d^Lifetime suicide plan and lifetime suicide attempt was determined by Suicidal Behavior Questionnaire-Revisited

SBQ-R score was significantly associated with lifetime depression history and current demoralization with SBQ-R scores being 1.6 and 2.8 points higher, respectively (Table 5). Chronic CH was marginally associated with a 1.1 point higher SBQ-R score (*p* = 0.056). The odds of passive or active SI in one’s lifetime was nearly seven-fold greater in those with demoralization. Again, having chronic compared to episodic CH was marginally associated with a higher odds of lifetime SI (*p* = 0.17). Lifetime depression was not associated with SI in CH sufferers.

**Table 5:** Associations of Suicidal Ideation and High Suicide Risk in Cluster Headache Patients

Outcome: Passive or Active Suicidal Ideation		
Variable	Odds Ratio (95% CI)	*p*-value
Chronic Cluster Headache^a^	2.03 (0.74, 5.57)	0.17
Lifetime depression^b^	1.89 (0.66, 5.46)	0.24
Demoralized^c^	6.66 (1.56, 28.49)	0.01*
Age (per 1 year)	0.98 (0.94, 1.02)	0.25
Male sex^d^	1.82 (0.60, 5.47)	0.29
Caucasian^e^	1.09 (0.21. 4.56)	0.99
Currently Married^f^	1.11 (0.34, 3.64)	0.87
Completed College^g^	0.43 (0.13, 1.43)	0.17
High/Middle Income^h^	0.97 (0.29, 3.29)	0.96
Regular Drug Use history^i^	1.15 (0.36, 3.66)	.081
Regular Alcohol Use history^j^	0.53 (0.08, 3.56)	0.51
Outcome: SBQ-R Score (Range 3-18)		
Variable	SBQ-R Score points (95% CI)	*p*-value
Chronic Cluster Headache^a^	1.1 (0.0, 2.2)	0.056
Lifetime depression^b^	1.6 (0.3, 2.8)	0.02*
Demoralized^c^	2.8 (1.5, 4.1)	0.0001*
Age (per 10 year)	0.1 (−0.3, 0.6)	0.63
Male sex^d^	−0.2 (−1.3, 1.0)	0.79
Caucasian^e^	0.1 (−2.1, 2.4)	0.91
Currently Married^f^	−0.2 (−1.3, 1.0)	0.78
Completed College^g^	−0.8 (− 2.1, 0.4)	0.20
High/Middle Income^h^	0.0 (−1.3, 1.2)	0.94
Regular Drug Use history^i^	0.6 (−0.6, 1.9)	0.31
Regular Alcohol Use history^j^	1.3 (−0.8, 3.4)	0.22

Reference groups: ^a^Chronic cluster headache: referent episodic cluster; ^b^Lifetime depression: referent no depression; ^c^Demoralization: defined as fulfilling demoralization diagnostic criteria for use in psychosomatic research or KDS score ≥ 42; ^d^Male gender: referent female; ^e^Caucasian: referent other races; ^f^Currently married: referent currently single, divorced, widowed; ^g^Completed college: referent did not complete collegel; ^h^High/middle income: referent low income (< $3000 monthly); ^i^Regular drug use: period of one year when drugs were used weekly, referenced to those that did not have such a period; ^j^History of regular alcohol use: period of one year when 3 or more drinks at least 3 times per week, referenced to those who did not have such a period

* = *p*<0.05

Of note, in controls, SI was associated with lifetime depression (OR 2.88 [1.16, 7.17]; *p* = 0.02), but not demoralization (OR 1.963 [0.57, 6.51]; *p* = 0.29). In contrast, SBQ-R score was associated with demoralization (2.84 [1.54, 4.13]; *p* = 0.0003), but not lifetime depression (0.50 [− 0.45, 1.46]; *p* = 0.30) in controls.

## Discussion

In this study specifically designed to study suicidality in CH, we found that persons with CH, in particular chronic CH, were significantly more likely to have had either passive or active SI in their lifetime than individuals without CH; furthermore, the likelihood of SI was best predicted by the presence of demoralization. We chose assess both depression and demoralization, as both phenomenon are important predictors of suicidality. Having high suicide risk was significantly more common among persons with CH compared to matched controls. The SBQ-R score was positively associated with having CH, a lifetime history of depression, and most highly with having a current state of demoralization. Our study corroborates previous research that also found high rates of suicidality in CH, but takes important additional steps. First, a control group which was carefully matched demographically and socioeconomically was used; second, questionnaires validated for the assessment of suicidality and mental health were utilized; and third, the phenomenon of demoralization which is particularly germane in suicidality was assessed. The inclusion of a control group enabled a determination of whether or not suicidality was associated with CH independent of sociodemographic and mental health factors. Given that previous studies lacked a control group, it remained unclear whether increased suicidality was attributed to CH or to another factor, such as depression, which is highly prevalent in CH. Our study found that suicidality was indeed associated with CH even when lifetime depression was considered. Demoralization is another factor influencing suicidality, but even after adjusting for demoralization, having CH was associated with suicidality. Demoralization more strongly predicted lifetime SI than lifetime depression in CH sufferers, while the reverse was true in control participants. Thus, the assessment of demoralization may be particularly important in persons with CH. These findings call attention to the enormous toll that CH takes on the mental health of patients, particularly in chronic CH sufferers who have no reprieve from attacks.

The recognition that CH is associated with suicidality has existed since the disorder’s initial descriptions by B.T. Horton in 1939 [6]. Later, CH patients began using the colloquial name, “suicide headache”, which imparts the excruciating nature of attack pain. Despite the early knowledge that CH is associated with suicidality, empiric evidence for high rates of SI were unavailable until recently with reports in 2011 and 2012 by Jurgens in Germany and Rozen in the United States, respectively [4, 20]. Jurgens found that one-quarter of CH patients had SI, while Rozen in a much larger sample found that 55% of CH patients had lifetime SI.

The results of our study showed that having CH, having a lifetime depression history, and being demoralized were significant predictors of having SI in one’s lifetime. In fact, demoralization increased the odds of lifetime SI more than three-fold, whereas lifetime depression increased lifetime SI two-fold. Furthermore, among those with CH, demoralization but not lifetime depression was associated with SI, with demoralization increasing the odds of lifetime SI more than six-fold. In controls, SI was associated with lifetime depression, but not demoralization, suggesting that demoralization may be particularly sensitive to suicidality in CH. The condition of demoralization is a psychological state characterized by feelings of helplessness, hopelessness, and subjective incompetence, a self-perceived incapacity to perform tasks deemed necessary in stressful situations [8, 21]. Although demoralization often co-occurs with depression, the two entities are distinct. In depression, the appropriate course of action is known, yet motivation to act is lacking, whereas in the demoralized state, there is prominent uncertainty as to the most suitable course of action [8]. Distinguishing between the two states is critical to formulate the best treatment plan, as demoralization may be best addressed with cognitive behavioral therapy by confronting maladaptive ways of thinking [22]. The absence of hope is central in demoralization. Hopelessness has been shown to correlate more strongly with SI than does depression [23].

Persons with CH are at increased risk of demoralization for a number of reasons. In general, chronic pain sufferers are at risk of demoralization [24], especially when hopelessness develops that there are no treatment options to decrease pain [25]. CH is often misdiagnosed; one large survey-based study found that proper CH diagnosis is delayed an average of 6.6 years [26]. Although there are treatments for CH and patients should not be left to feel that there are no options, some are not offered guideline-adherent treatment [27]. Furthermore, there are challenges in access to therapy; up to half of persons with CH rate having experienced some difficulty getting access to one of the most highly effective abortive agents for CH—high flow oxygen [28]. We found that those with chronic compared to episodic CH were more likely to be demoralized. One can imagine how a feeling of demoralization might emerge in those who receive no reprieve from intense paroxysms of pain. Chronic pain in general places sufferers at increased risk of both demoralization and SI [29]. While demoralization has not been previously assessed in persons with headache, SI and suicide attempts have been shown to be highly prevalent in migraineurs [30]. The association among migraine, depression, and suicidality is a complex one with a bidirectional association between migraine and depression [31].

Although suicide is often thought of as a consequence of depression, the situation in CH appears more complex. As reported by CH patients, they may assign an evil persona to their disease, such as “this beast” or “the devil” [32]. Anecdotally, patients describe the need to bring an end to this evil persona, with their own bodies as collateral. This incarnation of the disease and the depersonalization of self-harm do beg the question of whether SI in CH reflects the suicidality of major depression directed toward the self, or rather a desperate, destructive action directed toward the personified disease. In demoralization, there is uncertainty regarding the appropriate response to a perceived threat. As a result of this uncertainty and in desperation, the demoralized individual may misdirect their response toward suicide.

According to Joiner’s interpersonal theory of suicide, individuals with a thwarted sense of belonging, who perceive that they are a burden, may have a desire for suicide, but this differs from the capability to commit suicide [33]. According to the theory, the capability for suicide is acquired through the attainment of two essential components, increased pain tolerance and fearlessness of death. Over time, through the exposure to repeated painful events, one may experience an increased pain tolerance. When the pain is so great, a fearlessness of death may develop, culminating in the emergence of the capability for suicide [34]. The pain paroxysms in CH, while relatively short, are rated by CH sufferers as approximately 30% more severe than the pain of childbirth or nephrolithiasis, and CH sufferers report extreme fear and dread in anticipation of future attacks [3]. Needless to say, these painful repetitive events could result in an increased or at least perceived increased pain tolerance. At the same time, the sheer magnitude of suffering which occurs repeatedly could lead to a fearlessness about death in that the CH sufferer has already endured the most horrible pain known and herein may arise a perception that death may not be so terrible in comparison. Future studies of suicidality in CH should in addition to assessing depression and demoralization explore these concepts of pain tolerance, attitudes toward death (fearlessness), as well as whether SI is directed inwardly toward the self or outwardly toward the disease state. Sensory sensitivity is also associated with depression as both hypo- and hypersensitivity are associated with depression [35]. Altered sensory processing is also associated with impulsivity, another risk factor for suicide. The extent to which sensory and pain processing have been affected in CH ma mediate these important suicide risk factors and should be researched in future studies.

Our study has important strengths and limitations to consider. A major strength of our study is that we included a demographically and socioeconomically matched control group. Inclusion of this well-matched control group was critical in allowing us to demonstrate that SI was associated with CH, independent of such factors as depression. Furthermore, we used two validated and well-known questionnaires to assess suicidality, SBQ-R and C-SSRS, as well as standard ICHD-3 criteria to diagnose CH. It was important to assess lifetime depression as the aforementioned suicidality questionnaires delineate suicidality over a lifetime. In addition to evaluating depression, the psychological state of demoralization was studied, which may better characterize the desperation so often felt by CH patients. Limitations of this study include the relatively small sample size, its cross-sectional nature, and the assessment of prevalent depression with only self-report. A larger CH sample along with a longitudinal design would better discriminate the relationship of suicidal ideation to CH type and for episodic CH, whether or not SI is related to being in an active cycle. Pain and sensory processing were not assessed in the study, which are likely very pertinent predictors of suicide in pain disorders. Additionally, chronic CH patients comprised a larger proportion of CH cases than observed in the general CH population. This might be due to a greater desire among chronic CH patients to seek out CH research or CH support groups. The study also did not assess childhood traumatic experiences, an important risk factor for suicidality [36], nor did it assess perception of suicidal intent inwardly toward the self or outwardly toward the CH “beast”.

The results of this study uphold a long-held belief that patients with CH are at increased risk for suicide. Novel findings include the strong predictive power of demoralization for SI and suicide risk in CH, perhaps even more than in control participants. Future research is needed to determine whether the state of demoralization in CH patients is related to feeling that there are limited or ineffective treatments, that they have little or no access to providers who understand CH, or simply to the recurrent and severely painful nature of the disease. The key findings of this study that SI is increased in persons with CH and is related to both depression and demoralization highlight the importance of screening for not only suicidal ideation and intent, but also the psychological phenomenon of demoralization. It is critical that screening for SI, demoralization, and depression occur with regularity at every clinical encounter. This screening can be carried out rapidly by questionnaire prior to the visit. The DCPR-D consists of four questions (see methods) and can be rapidly administered in the clinic. Any depression screen administered should also include questions about SI. The presence of demoralization, depression, or SI should prompt a safety assessment, as well as psychiatric and psychological evaluations.

## Conclusions

Lifetime suicidal ideation and high suicide risk are prevalent in CH sufferers, significantly moreso than in sociodemographically matched control participants without CH. The odds of lifetime suicidal ideation were higher in those with CH than in controls, even after accounting for depression and demoralization. In CH, suicidal ideation was associated with demoralization but not depression. The presence of both suicidal ideation and demoralization should be screened for in patients with CH.

## Data Availability

The datasets generated during and/or analyzed during the current study are available from the corresponding author on reasonable request.

## Abbreviations

BLDS: Brief Lifetime Depression
CH: Cluster headache
CH-QOL: Cluster Headache Quality of Life Questionnaire
C-SSRS: Columbia Suicide Severity Rating Scale
DCPR-D: Diagnostic Criteria for use in Psychosomatic Research – Demoralization
ICHD-3: International Classification of Headache Disorders 3rd edition
KDS: Kissane Demoralization Scale
SI: Suicidal ideation
